# The Presence of Urinary Renal Progenitor Cells in Stable Kidney Transplant Recipients Anticipates Allograft Deterioration

**DOI:** 10.3389/fphys.2018.01412

**Published:** 2018-10-10

**Authors:** Anna Manonelles, Roser Guiteras, Edoardo Melilli, Elena Lazzeri, Montse Goma, Elena Crespo, Oriol Bestard, Anna Sola, Paola Romagnani, Josep M. Cruzado

**Affiliations:** ^1^Nephrology Department, L’Hospitalet de Llobregat, Hospital Universitari de Bellvitge, Barcelona, Spain; ^2^Experimental Nephrology, Department of Ciències Clíniques, Institut d’Investigació Biomèdica de Bellvitge (IDIBELL), Hospitalet de Llobregat, Universitat de Barcelona, Barcelona, Spain; ^3^Excellence Centre for Research, Transfer and High Education for the Development of DE NOVO Therapies (DENOTHE), University of Florence, Florence, Italy; ^4^Pathology Department, L’Hospitalet de Llobregat, Hospital Universitari de Bellvitge, Barcelona, Spain

**Keywords:** parietal epithelial cells, kidney transplantation, podocyte detachment, graft outcomes, estimated glomerular filtration rate, albuminuria

## Abstract

Long-term kidney transplant outcomes have reached mild improvements recently. Parietal epithelial cells (PECs) are progenitor cells located along the Bowman’s capsule that can be isolated in urine, and display the capability to replace podocytes, but in certain situations cause glomerulosclerosis. In this study, a cohort of stable kidney transplant recipients with 6 months protocol biopsy was divided in two groups depending on the presence (uPEC+; *n* = 41) or absence (uPEC-; *n* = 25) of PECs in urine and followed for 2 years. No differences were found between groups at 6 months after transplantation considering clinical variables, alloimmune response, renal function, albuminuria and graft pathology. However, uPEC+ group showed increased podocyturia and a higher rate of proliferating PECs along the Bowman’s capsule, without concomitant enhancement of the CD44 pro-sclerotic activation marker. Accordingly, 2 years follow up evidenced poorer outcomes in the uPEC+ group with worse renal function, increased albuminuria, wider mesangial expansion and more severe IFTA. In summary, chronic allograft damage can progress in certain stable-supposed grafts by podocyte detachment and reactive PECs proliferation, being the uPEC presence a biomarker of this process. This damage-response regenerative process, if sustained in time, might fail in preserve the allograft function and histology. Our study raises new prospects to overcome current limits on long-term allograft results.

## Introduction

Chronic kidney disease is a leading cause of morbidity and mortality nowadays, with a renal replacement therapy (RRT) prevalence of 924 patients per million population in Europe (Pippias et al., 2017). Kidney transplantation is the RRT option that offers the best outcomes in terms of life expectancy and quality of life (Wolfe et al., 1999). In this sense, new approaches are needed to improve graft function and survival especially considering long-term outcomes, where clinical advances have been subtle in the last years (Meier-Kriesche et al., 2004; Nankivell and Kuypers, 2011; Riella et al., 2016).

Many studies have been performed focusing on the mechanisms of kidney graft damage, especially on ischemia-reperfusion injury, alloimmunity, nephrotoxicity and disease recurrence (Briganti et al., 2002; Menke et al., 2014; Bamoulid et al., 2015), but little is known about the own mechanisms the kidney uses to compensate these damages and the potential regenerative processes behind.

The recently identified progenitor cell population surrounding the Bowman’s capsule displays a multipotent capacity of differentiation into kidney specific cells terminally differentiated (podocytes and tubular cells), which are characterized by the co-expression of two species- specific markers, CD133 and CD24 (Sagrinati et al., 2006). These Parietal Epithelial Cells (PECs) exhibit the capacity to ameliorate acute kidney damage (Lasagni et al., 2015), but in certain conditions these cells might contribute to crescent formation (Sicking et al., 2012) or glomerulosclerosis by characteristically *de novo* expression of CD44 (Smeets et al., 2011) or by undergoing epithelial to mesenchymal transition (Naito et al., 2014).

PECs are eliminated in urine in certain physiological and pathological conditions and have already been used to perform functional and genotypic studies without the need of invasive procedures (Achenbach et al., 2008; Lazzeri et al., 2015; Arcolino et al., 2016).

Investigations on kidney allograft deterioration are mainly focused on mechanism of allograft damage without considering their opponents, the intrinsic mechanisms of kidney repair. Among such mechanisms, PECs may have a relevant role on modulating the impact of any injury.

Therefore, in our study we evaluated the presence of PECs in the urine of a cohort of stable kidney transplant recipients at 6 months after transplantation in order to elucidate its association with pathological findings (Haas et al., 2014) in protocol biopsies as well as immunological (Bestard et al., 2013) and non-immunological mechanism of allograft damage. We then investigated the association of PECs in the urine with kidney allograft function and pathology at 2 years after kidney transplantation.

We consider regeneration the cornerstone of novel kidney transplant care, raising potential new prospects for future prognostic features and therapeutic targets.

## Materials and Methods

### Study Population and Urine Samples

This is a prospective cohort study approved by Bellvitge Hospital Institutional Review Board (approval reference PR075/12). After written informed consent, kidney transplant recipients with stable renal function were prospectively included in the study from 2013 to 2015 at 6 months after transplantation, at the time of hospitalization due to the surveillance biopsy procedure according to our regular clinical practice. Sample contamination, defined as visual fungi or bacterial overgrowth in cell culture within 24–72 h, was an exclusion criterion. Patients were followed for 2 years when a second kidney allograft biopsy was planned.

### Standardized Protocol for the Isolation and Purification of Urine Parietal Epithelial Cells

Urine samples were treated with Penicillin-Streptomycin Solution (Biological Industries, Cromwell, CT, United States) at 1:100 dilution, and then were centrifuged at 1500 rpm for 10 min as previously described (Lazzeri et al., 2015). The supernatant was carefully removed, and the cell pellet was washed with PBS (Dulbecco’s PBSLinus, Sigma-Aldrich, St. Louis, MO, United States) and centrifuged again at 1500 rpm for 5 min. The supernatant was then removed and the cell pellet was plated in 6-well dishes with EGM-MV media (Lonza Sales Ltd.) supplemented with 20% FBS (Hyclone Laboratories, South Logan, UT, United States) and antibiotics (Pen-Strep + rifampicin). Twenty-four hours after the culture, medium was changed in order to remove all the unattached cells and other detritus. Only cells attached to culture plates were tributary to study. After the 1st week it was possible to identify by optical microscopy attached cells, which achieved 80% confluence in about 2–3 weeks. It was possible to marginally identify other attached cell types like fibroblasts or podocytes. Medium was changed twice a week, and no cell passaging was performed. In order to confirm their clonogenicity, control cells were cloned and maintained in culture without incidences, and it was possible to expand them and perform different culture passages kept in undifferentiated state.

### Fluorescence Activated Cell Sorting

At 80% confluence, adherent cells were removed from 6-well plates (35 mm diameter) by incubation in 0.05% trypsin–EDTA (Life Technologies, Bleiswijk, Netherlands) at 37°C, and were subjected to Fluorescence Activated Cell Sorting (FACS) at passage 0 in order to specifically select those cells positive for glyCD133 and CD24. For all experiments, the cells were stained with CD24-PE (ML5, BD Pharmigen, San Diego, CA, United States) and CD133-APC (clone 293C3, MiltenyiBiotec monoclonal) antibodies for 30 min at room temperature. Unstained cells and FMO (fluorescence minus one), treated in the same way, were used as negative control in each experiment and served as autofluorescence control. The control isotypes used were for CD133 mouse IgG2b-APC (clone IS6–11E5.11) (MiltenyiBiotec GmbH), and for CD24 mouse IgG1-FITC (clone IS5–21F5) (BD Pharmigen, San Diego, CA, United States). Cell death was evaluated using DAPI staining (D9542-1MG, Sigma-Aldrich, St. Louis, MO, United States). Cells were filtered through a 40 μm strainer to eliminate all remaining cell clumps. Sorting was done using a MoFloAstrios cell sorter (Beckman–Coulter) equipped with a 100 μm flow tip and operated at a sheath pressure of 25 psi. Initially, dead cells and debris were gated on a two physical parameter dot plot (FSC/SSC) followed by the exclusion of doublets by using pulse processing (FSC-H vs. FSC-A). Dead cells were excluded (DAPI+). Finally, the CD133 + CD24 + subpopulation was gated. An average sorting rate of 500–1000 events per second was maintained Cells were >98% pure after sorting. All sorted cells were frozen in dry pellet and stored in 100,000 cells vials at -80°C until use.

### Urinary Parietal Epithelial Cells *in vitro* Characterization and Differentiation

Urinary PECs (uPECs) obtained from cell sorting were kept in culture at 1st passage in order to perform differentiation into terminal renal cells to confirm their multipotent capacity. As previously described (Lazzeri et al., 2015) for the differentiation toward the tubular lineage, uPECs (*n* = 5) were cultured in the tubular differentiating medium renal epithelial cell growth medium (Lonza Sales Ltd.) supplemented with 50 ng/mL of hepatocyte growth factor (Peprotech, Rocky Hill, NJ, United States) for 21 days. For the differentiation toward podocytes, uPECs (*n* = 5) were cultured in the podocyte- differentiating medium VRAD, composed of DMEM/F12 (Sigma-Aldrich, St. Louis, MO, United States) supplemented with 10% FBS (Hyclone), 100 nM vitamin D3, and 100 mM retinoic acid, for 48 h (all from Sigma-Aldrich, St. Louis, MO, United States). Confocal microscopy was performed on differentiated and non-differentiated cells to identify podocyte, tubular and cytoskeleton markers. The following antibodies were used for the detection of tubular antigens: anti-Aquaporin1 (H-55, Santa Cruz Biotechnology, Santa Cruz, CA, United States), anti-LTA (Fluorescein Vector Laboratories, Burlingame, CA, United States), anti-DBA LTA (Rhodamine, Vector Laboratories, Burlingame, CA, United States). Podocyte markers were Anti-Podocin (NPHS2, Abcam, Cambridge, United Kingdom), Anti-Podocalyxin (PDLX, R&D Systems, Canada). Cytoskeleton staining was performed with Alexa Fluor 555 Phalloidin (Molecular Probes from Invitrogen). To-pro-3 iodide (642/661 Molecular Probes Invitrogen detection Technologies) was used for counterstaining nuclei. Secondary antibodies included for AQP1 Alexafluor 488 goat anti rabbit (Invitrogen), for NPHS2 Alexafluor 488 goat anti rabbit (Invitrogen) and for PODXLAlexafluor 488 goat anti mouse (Invitrogen).

### Kidney Allograft Biopsies

Protocol biopsies were taken at 6 and 24 months after kidney transplantation, where two core samples were obtained under ultrasound guidance. There were no major complications related to the procedure. Renal slices were fixed in 10% (vol./vol.) formalin and embedded in paraffin. Histological cross sections of 3 μm thickness were stained with haematoxylin and eosin, periodic acid–Schiff and Masson’s trichrome for optical microscopy assessment. The biopsies were blindly evaluated by a pathologist (Dr. Gomà) using the Banff 2013 classification (Haas et al., 2014).

The CD133 staining was performed with CD133/2 (293c3) APC monoclonal mouse anti human (1:2.5; MilenyiBiotec, Germany), with secondary antibody goat anti-mouse IgG2b- 488 (Invitrogen, Alexa Fluor) and evaluated by confocal immunofluoresence. CD44 antibody was monoclonal mouse anti human (1:100; 156-3c11, Abcam, Cambridge, United Kingdom), with goat anti-mouse IgG2A HRP (Novus Biologicals, Abingdon Oxon, United Kingdom), and evaluated by immunohistochemistry. We evaluated the Bowman’s capsule epithelium and the glomerular tuft, counting the number of CD44+ cells and then calculating the mean of CD44+ cells per glomerulus. Immunohistochemistry was also performed for Ki67 (1:20; BD Pharmingen, San Diego, CA, United States). We counted positive cells on Bowman’s capsule epithelium and glomerular tuft, normalized by the total glomerulus number in the sample.

### Clinical and Analytical Data

Donor demographics were evaluated including age and gender, as well as its characteristics being living or deceased donor, and extended criteria donors (ECD: age 60 years or older, or over 50 years with at least two of the following conditions: hypertension history, serum creatinine >1.5 mg/dL or cause of death from cerebrovascular event). Variables evaluated from the recipient included: age, gender, end-stage renal disease (ESRD) etiology, time in dialysis, residual urinary volume, human leucocyte antigen (HLA) mismatches, anti-HLA antibodies (Panel Reactive antibodies >20%) and biological relation between donor and recipient. Variables related to the transplantation procedure included cold ischemia time and history of delayed graft function (defined as the need of hemodialysis during the first 7 days after kidney transplantation). Regarding immunosuppression protocols, basiliximab was used for regular recipients and thymoglobulin in high-immunological-risk and ECD recipients. We considered high-immunological risk recipients those that had lost a previous graft due to immunological reasons during the 1st year after transplantation, those with any anti-HLA donor specific antibody and those with a cytotoxic panel reactive antibody (PRA) > 20%. Steroids, calcineurin inhibitors (tacrolimus in 64 patients), and mycophenolate mofetil were given to all patients. Maintained immunosupression treatment at surveillance biopsy time-point included steroid withdrawal (*n* = 5) and conversion from mycophenolate mofetil to mTOR inhibitors (*n* = 5). Analytical data considered estimated Glomerular Filtration Rate (measured with CKD-EPI formula), proteinuria and albuminuria at 3, 6 months, 1 and 2 years after transplantation, tacrolimus average levels, vitamin D levels and glycemia at the biopsy moment. Clinical variables were recorded including body mass index (BMI), body surface area (BSA, by Dubois formula), hypertension and diabetes diagnosed at the time of the 6 months biopsy, as well as history of acute rejection events. Blood pressure measurements at 6 months after kidney transplantation were determined after 10 min rest and with three measures, using the median value of them for analysis. In case of white coat effect, home blood pressure monitoring was used. Concomitant medications as renin-angiotensin system blockers and other anti-hypertensive drugs, insulin and oral antidiabetic drugs, and vitamin D supplements were recorded.

### Immunological Data

Immunological tests considered the presence of circulating anti-HLA class I and II alloantibodies, performed in banked serum samples prior to kidney transplant and at 6 months after it (at the moment surveillance biopsy was performed). Antibody specificities against both class I and II HLA antigens were determined using single-antigen flow beads assays on a Luminex platform (Lifecodes, Division of Immucor, Stanford, CT, United States). All beads showing a normalized MFI > 1500 MFI were considered positive if (MFI/(MFI lowest bead)) > 5.

Peripheral blood mononuclear cells (PBMCs) were obtained in heparinized tubes from renal transplant patients pre-transplantation and at the time of 6 months protocol biopsy.

Donor cells were harvested before transplantation from donor spleens or peripheral blood samples in deceased or living donors, respectively. PBMCs and splenocytes were isolated by standard Ficoll density gradient centrifugation and were frozen in liquid nitrogen for subsequent use in the IFN-γ ELISPOT assays.

Donor-specific (d-s) IFN-γ ELISPOT assays were performed following recently described standard operating procedures (SOP) (Bestard et al., 2013). Briefly, 3 × 10^5^ responder cells were placed in triplicate wells with 3 × 10^5^ CD2-depleted splenocytes (Easysep^®^ Human CD2 Selection kit, StemCell, France) or CD3-depleted living-donor PBMCs (human CD3+ Cell Depletion Cocktail, RosetteSep^®^ kit, StemCell, France). Recipient cells were stimulated with complete medium alone (RPMI 1640, GE Healthcare Life Sciences, United States, with 10% inactivated FBS, antibiotics and L-glutamine) and Pokeweed (AID, Autoimmune Diagnostika) as negative and positive controls, respectively. Results were given as frequencies of IFN-γ producing d-s T-cells/3 × 10^5^ PBMCs, subtracting responses of the negative control wells.

### Gene Expression Profile

It was previously described that PECs obtained from urine exhibit an almost identical transcriptome compared to those obtained from kidney tissue (Lazzeri et al., 2015). We then performed Quantitative real-time PCR to selected molecules that might suffer changes in kidney damage conditions for PECs, like CXCL12, that mediates stem cell niche maintenance through its receptors by homing and differentiating PECs in kidney damage, TLR2 that plays a role in the activation, proliferation rate and differentiation of PECs by autocrine signaling. We also analyzed collagen IV and αSMA to evaluate extracellular matrix production and Epithelial-to-Mesenchymal transition, CD44 for glomerulosclerotic pattern. Vascular cell adhesion molecule 1 also as progenitor cell commitment pattern, Wnt7b has a specific role in controlling renal morphogenesis and in tubule recovery by activating Wnt signaling pathways.

For Quantitative real-time PCR, RNA was extracted from kidney (*n* = 3) and urine (*n* = 8) PECs with Pure Link RNA Mini Kit (Invitrogen, CA, United States), using a Trizol reagent (Invitrogen, CA, United States) to lyse the tissues, chloroform to separate the organic and aqueous phase (where the RNA remains) and the ethanol to purify the RNA. RNA purity was analyzed on a NanoDrop (NanoDrop ND-1000V3.3, Wilmington, DE, United States) and was considered pure when the absorbance ratio 260/280 nm was higher than 1.80. A total amount of 50 ng RNA was used to perform the reverse transcription using a High-Capacity cDNA Reverse Transcription Kit (Applied Biosystems, Warrington, United Kingdom). Thermal cycling conditions were 10 min at 25°C, 120 min at 37°C, 5 min at 85°C and finally held at 4°C. Tissue and uPECs expression levels of CXCL-12, TLR-2, COL-IV, α-SMA, CD44, VCAM, and Wnt7b were quantified by TaqMan real-time PCR (ABI Prism 7700, Applied Biosystems, Waltham, MA, United States) using the comparative 2^-ΔΔC_t_^ method (Applied Biosystems).

### Urinary Human Podocin Quantification

Urine samples were collected from all patients included in the study. Samples were selected and centrifuged for 30 min at 3000 g, and supernatant was properly aliquoted and stored at -80°C. Quantitative assessment of Podocin (OKEH00672) protein was carried out in urine samples by enzyme-linked immunosorbent assay (ELISA) (Aviva Systems Biology, San Diego, CA, United States) following the manufacturer’s recommended instructions. Quantification of human Podocin was calculated using the standard curve.

### Statistics

Continuous variables with normal or symmetrical distribution were reported as mean and standard deviation above and below the mean. The categorical variables were described with frequencies and percentages. The differences between the two groups for continuous variables were analyzed using Student’s *t*-test or the Wilcoxon rank-sum test, as appropriate. The differences between categorical variables were analyzed using the chi-squared distribution and Fisher’s exact test, as appropriate. ANCOVA was performed to evaluate the relationship between a variable and a covariate. All statistical tests were considered significant if the *p*-value was <0.05 for two-tailed tests. The statistical analysis was performed with the Stat View SAS program.

## Results

### Isolation of Parietal Epithelial Cells From Urine of Kidney Allograft Recipients

**FIGURE 1:**
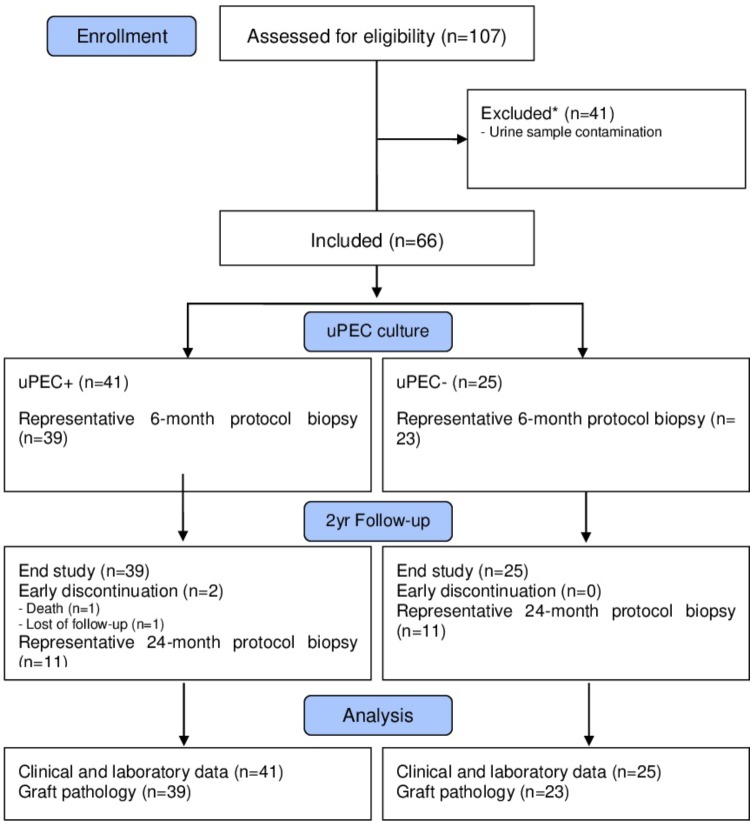
Flow diagram of the study population. We included stable kidney allograft recipients at 6 m after transplantation when a protocol kidney biopsy was performed. Over 66 graft biopsies, there were 11 missing values due to no representative sample (*n* = 7) or other complications (*n* = 4). Patients were then classified according presence of absence of uPECs and followed for 2 years.

**FIGURE 2:**
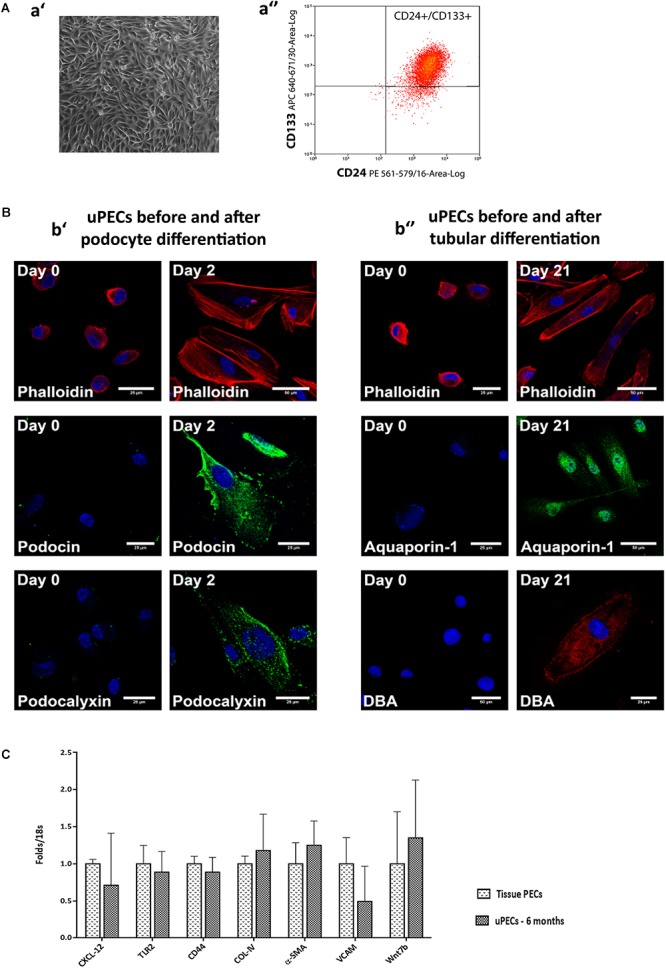
Characterization of urinary Parietal epithelial cells. **(A)** Phenotypical characteristics of uPECs **(a′)** Optical microscopy image of representative sample of 20 days cultured uPECs, in its characteristic rose-shape manner, at 400×. **(a′′)** Flow cytometry analysis, with CD133^+^ CD24^+^ subpopulation gated. **(B)** Functional and phenotypical characteristics of uPECs. Representative confocal microscopy uPEC cultures before and after differentiation into Podocytes and Tubular Cells; scale bar = 25 μm. **(b′)** Expression of podocyte markers (podocin, podocalixin) before (day 0) and after (day 2) differentiation medium VRAD (*n* = 5). **(b′′)** Expression of tubular markers (aquaporin- 1, DBA) before (day 0) and after (day 21) differentiation medium ECGM (*n* = 5). Every cell type cytoskeleton was evaluated by phalloidin staining. DAPI counter stains nuclei (blue). One representative sample is shown over five samples processed for each differentiation. **(C)** mRNA expression profile comparing uPECs to renal tissue PECs. For Quantitative real-time PCR, PECs RNA was extracted from kidney (*n* = 3) and urine (*n* = 8) PECs with Pure Link RNA Mini Kit (Invitrogen, CA, United States). *p* = NS by Mann–Whitney test.

After written informed consent, we included 107 patients prospectively in whom 6 months protocol biopsy was performed and urine samples were collected, where uPEC isolation protocol was performed (**Figure 1**). In 41 out of 66 patients, it was possible to identify single flat body epithelial cells growing into clusters in a rose shape manner from the 1st week after plating, as uPECs were undetectable by cytospins directly from urine due to its low number. These cells achieved a confluence of 80% in 35 mm diameter plates at 2–3 weeks after culture and more than 98% co-expressed CD133^+^CD24^+^ (**Figure 2A**).

### Cultured uPECs Exhibit Phenotypical and Genotypical Characteristics of Renal Progenitors

Urinary CD133^+^CD24^+^ cells were negative for podocyte (podocin, podocalyxin) and tubular epithelial cell (aquaporin 1, LTA, DBA) markers visualized by confocal microscopy. To evaluate the multipotency of uPECs into terminal kidney cells, uPECs were cultured under differentiation medium and evaluated by confocal microscopy. After differentiation into podocytes they expressed markers such as podocin (NPHS2) and podocalyxin (PODXL), with a characteristic sun shape on the cytoskeleton phalloidin evaluation. When differentiated into tubular cells, they expressed aquaporin 1 (AQP1) and DBA (**Figure 2B**). Although in a previous study we described that PECs obtained from urine exhibit an almost identical transcriptome compared to those obtained directly from kidney tissue (Lazzeri et al., 2015), we sought to further compare uPECs and kidney PECs gene expression profile regarding molecules related to stem cell recruitment, proliferation and activation (CXCL-12, TLR-2), pro-sclerotic and fibrotic pattern molecules (CD44, COL-IV, and α-SMA) and progenitor cell commitment patterns (VCAM, Wnt7b). Notably, no significant differences were found comparing PECs isolated from urine and kidney PECs (**Figure 2C**).

### No Donor and Transplant Recipient Characteristics Are Found to Be Associated to the Presence of uPECs at 6 Months After Transplantation

We then assessed whether the isolation of uPECs was associated with some donor or recipient baseline characteristics. As showed in **Table 1**, no associations were found. Pointedly, no significant differences were evidenced regarding immunosuppression therapy, neither in induction (basiliximab or ATG) nor in maintenance therapy.

**Table 1:** Kidney transplant baseline characteristics.

	uPEC+	uPEC-	*P*-value
	*n* = 41	*n* = 25	
Donor type (D/L)	26/15	14/11	0.61
Donor age (yr)	51.2 ± 13.4	54.5 ± 9.0	0.27
Donor gender (M/F)	25/16	12/13	0.32
Recipient age (years)	48.2 ± 13.6	53.5 ± 9.4	0.09
Recipient gender (M/F)	31/10	14/11	0.11
BMI (kg/m^2^)	25.5 ± 5.0	26.8 ± 4.8	0.33
Dialysis time (m)	19.2 ± 27.8	19.5 ± 23.8	0.96
Cause of ESRD	5	2	0.69
Diabetes	8	8	
Glomerular disease	7	1	
Hereditary	8	5	
Hypertension	7	5	
Interstitial	6	4	
Unknown			
Previous transplant (Y/N)	5/36	6/19	0.46
HLA mismatch	3.8 ± 1.4	3.6 ± 1.4	0.66
AntiHLA Ab (POS/NEG)	7/34	8/17	0.27
HLA-DSA (POS/NEG)	1/40	0/25	0.99
T-cell ELISPOT	52 ± 55	55 ± 57	0.86
Induction (BAS/ATG)	31/10	18/7	0.78

*D, deceased; L, living; M, male; F; female; BMI, body mass index; ESRD, end stage renal disease; HLA, human leukocyte antigen; Ab, antibody; DSA, donor specific antibody.*

Moreover, no differences were observed in key outcome variables such as acute rejection, delayed graft function, proteinuria, albuminuria and the renal function achieved at 6 m after kidney transplantation (**Table 2**). Also, no significant differences were found considering hypertension, neither in terms of prevalence nor in median values of systolic and diastolic blood pressure, obesity, diabetes and serum calcidiol levels (**Table 2**). The use of different therapies such as insulin, oral antidiabetics, vitamin D supplementation, and the number of anti-hypertensive drugs required and specifically ACE inhibitors (only five patients were receiving this treatment) were also similar between the uPEC + and uPEC- groups. All patients were under a CNI-based regimen, without significant differences in the average tacrolimus level achieved (uPEC + 6.2 ng/mL; uPEC- 6.32ng/mL, *p* = 0.79).

**Table 2:** Significant data and outcomes at 6 months after kidney transplantation.

	uPEC+	uPEC-	*P*-value
	*n* = 41	*n* = 25	
DGF (Y/N)	7/34	3/22	0.73
Acute rejection (Y/N)	3/38	0/25	0.28
Corticosteroids (Y/N)	38/3	23/2	0.99
Hypertension (Y/N)	18/23	7/18	0.29
RAS Blockade (Y/N)	4/37	1/24	0.64
BMI (kg/m^2^)	25.8 ± 4.7	27.2 ± 4.5	0.25
Diabetes (Y/N)	6/35	5/20	0.11
Calcidiol (ng/mL)	56.8 ± 26.4	40.0 ± 20.1	0.25
Vitamin D/VDRA (Y/N)	13/28	8/17	0.99
eGFR (CKD-EPI) mL/min/1.73 m^2^	56.0 ± 16.5	59.3 ± 19.1	0.47
Proteinuria (g/mol)	16.1 ± 12.7	13.2 ± 13.4	0.38
Albuminuria (g/mol)	7.1 ± 10.2	3.3 ± 5.8	0.11
HLA-DSA (Y/N)	3/38	2/23	0.55
T-cell ELISPOT	28 ± 70	20 ± 19	0.62

*DGF, delayed graft function; RAS, renin angiotensin system; BMI, body mass index; VDRA, vitamin D receptor agonist; eGFR, estimated glomerular filtration rate; HLA-DSA, human leukocyte antigen-donor specific antibodies.*

### Immunological Monitoring and Histological Evaluation According to Presence or Absence of uPECs

Immunological response against the allograft was evaluated by assessing peripheral B and T-cell alloreactivity and kidney graft pathology. We evaluated the presence of circulating anti-HLA class I and II alloantibodies, donor specific antibodies and also the presence of memory T-cells (Bestard et al., 2013) prior to kidney transplantation and at 6 months thereafter and no significant differences were found depending on the presence or absence of uPECs (**Tables 1, 2**). Six-months graft biopsies showed no significant histological changes on separate items (**Table 3**), occurring one episode of subclinical rejection in each group (type IA on negative uPEC group, IIB on uPEC + group) and borderline changes in 6 patients. No differences on chronic renal lesions were found considering also glomerular sclerosis, mesangial expansion, and IFTA. Two cases showed C4d positive staining without other antibody mediated rejection (ABMR) feature, both in the uPEC + group. Three patients developed *de novo* DSA at 6 m without ABMR changes on the biopsy (2 of them from uPEC- group). No differences between groups were found related neither to immunoglobulin’s deposition, and no cases of disease recurrence were evidenced.

**Table 3:** Kidney allograft histology according to Banff items in 6 months protocol biopsies.

	uPEC+	uPEC-	*P*-value
	*n* = 39	*n* = 23	
ag (0/1/2/3)	35/4/0/0	21/1/1/0	0.312
ai (0/1/2/3)	33/6/0/0	15/7/0/1	0.138
at (0/1/2/3)	31/6/1/1	18/2/3/0	0.313
ti (0/1/2/3)	26/13/0/0	12/10/0/1	0.299
ptc (0/1/2/3)	38/0/1/0	23/0/0/0	0.433
av (0/1/2/3)	38/0/1/0	23/0/0/0	0.433
aah (0/1/2/3)	32/6/1/0	20/2/1/0	0.695
cg (0/1/2/3)	38/1/0/0	22/1/0/0	0.377
ci (0/1/2/3)	28/10/1/0	16/6/1/0	0.935
ct (0/1/2/3)	29/9/1/0	16/7/0/0	0.642
cv (0/1/2/3)	35/3/1/0	19/3/1/0	0.514
mm (0/1/2/3)	36/3/0/0	20/1/2/0	0.164
C4d (POS/NEG)	2/37	0/23	0.526

### CD133+ PECs From Bowman’s Capsule Express CD44 Independently of uPEC Isolation

**FIGURE 3:**
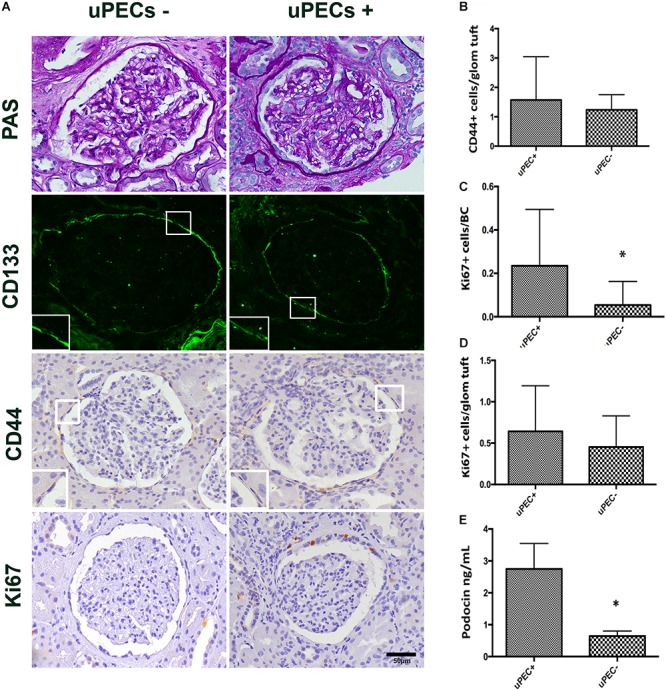
Immunophenotypic glomerular characterization and urinary Podocin at 6 months protocol biopsies. **(A)** Representative glomerular pictures on glomerulosclerosis evaluation, where no differences in glomerular lesions were found between groups; localization of PECs surrounding the Bowman’s capsule by CD133 + immunofluorescence; CD44 and Ki67 expressing cells evaluated along the Bowman capsule and the glomerular tuft; magnification 400×, scale bar = 50 μm (*n* = 22 for uPEC-, and *n* = 36 for uPEC +) **(B)** CD44 expression per glomerular profile was not different between groups (*n* = 22 for uPEC-, and *n* = 36 for uPEC+, *p* = NS); **(C)** no differences in Ki67 expression per glomerular profile were found (*p* = NS); **(D)** In uPEC+ group Ki67 was significantly increased in PECs along the Bowman’s capsule (*p* = 0.04). **(E)** Urinary podocin was significantly higher in the uPEC + group (*p* = 0.04). ^∗^*p* < 0.05.

We confirmed by immunofluorescence on 6 months protocol graft biopsies that PECs expressed the characteristic CD133^+^ marker all along the Bowman’s capsule (**Figure 3A**). To evaluate if these cells were activated according to previously described (Smeets et al., 2014), we performed CD44 staining in paraffin slides from all biopsies, showing no significant differences between uPEC+ and uPEC- groups (*p* = 0.26) (**Figure 3B**).

### The Presence of uPECs Is Associated With PECs Proliferation in the Bowman’s Capsule and With Podocyturia

We evaluated the presence of glomerular proliferating cells by the Ki67 marker that states active phases of the cell cycle by immunochemistry in graft biopsies (Wharram et al., 2005; Andeen et al., 2015; Nishizono et al., 2017). The number of Ki67^+^ PECs along Bowman’s capsule per glomerular profile was significantly increased in graft biopsies of uPEC+ compared to those uPEC- patients (**Figure 3C**). Of note, no differences were found in Ki67 expression in the glomerular tuft (**Figure 3D**). Podocyte loss was evaluated by assessing the concentration of urinary podocin (UPod) (Yang et al., 2015; Gilani et al., 2017) at the time of the biopsy. Interestingly, the uPod was significantly higher in the uPEC+ than in the uPEC- group (**Figure 3E**).

### The Presence of uPECs at 6 Months After Kidney Transplantation Is Associated With eGFR Decline, Albuminuria, and Glomerulosclerosis at 2 Years After Kidney Transplantation

We performed a 2 years follow-up of the patients included in the study, when a second protocol biopsy was performed. One patient was lost at follow-up and another died due to sudden death, both in the uPEC+ group. Thus, 64 out of 66 cases were available for the 2 years analysis.

**FIGURE 4:**
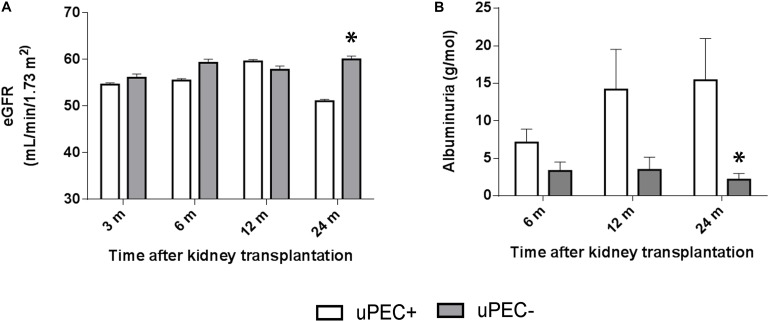
Estimated glomerular filtration rate and albuminuria evolution. Evaluation performed at 6 months, 1 and 2 years in uPEC+ and uPEC- groups. A significant decline in graft function (*p* = 0.04) **(A)** and increased albuminuria (*p* = 0.03) **(B)** is evidenced in the uPEC+ group at long-term follow up. ^∗^*p* < 0.05.

As showed in **Figure 4**, the uPEC+ group displayed a significantly lower eGFR and higher proteinuria and albuminuria at 2 years. In concrete, mean eGFR was 51.05 mL/min in uPEC + patients vs. 60.0 mL/min in uPEC- ones (*p* = 0.04). After adjustment for baseline (6 months) eGFR, the 2 years eGFR difference between uPEC+ and uPEC- groups was 8.95 mL/min and remained statistically significant (*p* = 0.01). Moreover, higher proteinuria and albuminuria was evidenced in the uPEC+ group (uPEC+ patients 15.4 g/mol vs. 2.1 g/mol in uPEC- ones, *p* = 0.03) at 2 years than the uPEC- group. Indeed, the values of albuminuria at 6 and 12 months after transplantation were normal in most patients either from uPEC+ or uPEC- group. However, at 2 years the proportion of patients with albuminuria was 17.4% in uPEC- and 48% in uPEC+ (*p* = 0.03). Results evaluated after adjusting for baseline albuminuria remain statistically significant.

**FIGURE 5:**
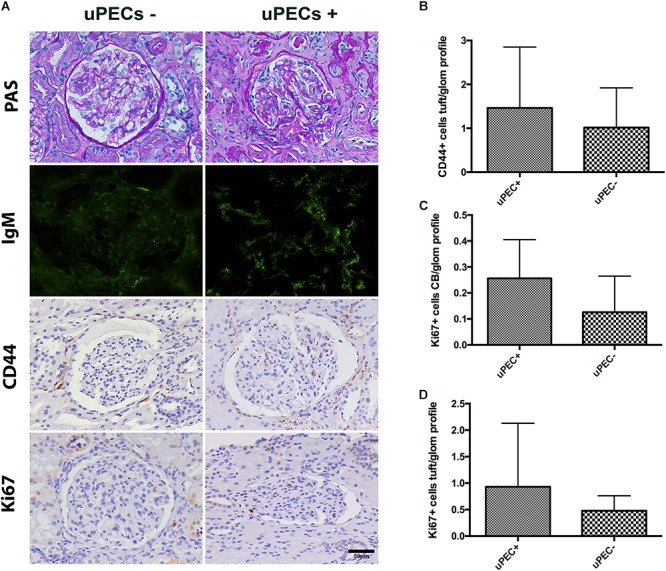
Evaluation of 24 months protocol biopsies. **(A)** An increased extracellular matrix deposition is evidenced in the uPEC+ group evaluated by PAS analysis and also a significant deposition of IgM in a glomerular focal and segmental pattern (*p* = 0.025); magnification 400×, scale bar = 50 μm. **(B)** CD44 expression per glomerular profile was similar between groups, as well as along the Bowman’s capsule. **(C)** A tendency of increased proliferation assessed by Ki67 along the Bowman’s capsule is still evidenced, while no differences are found in proliferation in the glomerular tuft **(D)**.

These results were in concordance with the histological findings observed in the available 24 months protocol biopsies (**Table 4**). Indeed, uPEC+ patients showed more mesangial matrix expansion (mm Banff score was significantly higher in uPEC+ group, *p* = 0.035) (**Table 4**) and IgM deposition (70% in uPEC+ group, compared to 20% deposition in uPEC- group, *p* = 0.025) (**Figure 5A**). No cases of primary glomerular disease recurrence were evidenced.

**Table 4:** Kidney allograft histology according to Banff items in 24 months protocol biopsies.

	uPEC+	uPEC-	*P*-value
	*n* = 11	*n* = 11	
ag (0/1/2/3)	9/2/0/0	8/3/0/0	0.611
ai (0/1/2/3)	7/3/1/0	8/2/1/0	0.875
at (0/1/2/3)	8/3/2/0	9/1/1/0	0.822
ti (0/1/2/3)	6/3/2/0	7/3/1/0	0.851
ptc (0/1/2/3)	10/1/0/0	10/1/0/0	1.000
av (0/1/2/3)	11/0/0/0	11/0/0/0	1.000
aah (0/1/2/3)	6/3/2/0	6/3/0/0	0.319
cg (0/1/2/3)	11/0/0/0	11/0/0/0	1.000
ci (0/1/2/3)	4/4/3/0	5/6/0/0	0.173
ct (0/1/2/3)	3/6/1/1	4/7/0/0	0.528
cv (0/1/2/3)	7/3/1/0	9/2/0/0	0.484
mm (0/1/2/3)	6/5/0/0	11/0/0/0	0.035
C4d (POS/NEG)	0/11	0/11	1.000

Finally, we evaluated if PEC proliferation was maintained or predominant activated PECs were present at 24 months after transplantation. Similar results emerged regarding CD44 expression, where no differences were found between groups (**Figure 5B**). A tendency of increased proliferation (Ki67+) on the Bowman’s capsule from uPEC+ patients was evidenced compared to uPEC- group (**Figure 5C**), while no proliferative differences were found in the glomerular tuft (**Figure 5D**).

## Discussion

Progressive kidney transplant attrition has become a difficult obstacle to overcome, as no significant improvements have been achieved in the last years. In this sense, new approaches are needed (Nankivell and Kuypers, 2011). Besides immunological issues, the inner repair graft capacity could be a game changer in generating new hypothesis and therapeutic targets. Actually, the study of parietal epithelial cells (PECs) in kidney transplantation may open new prospects in terms of kidney adaptation and damage response.

In our study, we demonstrated for a first time that kidney allograft PECs are excreted and can be isolated from the urine in a subgroup of stable kidney transplant recipients at 6 months after transplantation independently to demographic and transplant characteristics, and without association to alloimmune effector T and B cell mechanisms. The presence of PECs in the urine 6 months after transplantation was associated with podocyte loss and glomerular PEC proliferation in the allograft and what is more important, with poor graft outcome as demonstrated by eGFR decline, proteinuria and chronic allograft damage after 2 years follow-up.

Based on our previous experience in uPEC isolation (Lazzeri et al., 2015) we tried to ascertain whether uPECs were present in the urine after kidney transplantation. During the 1st week after transplantation PECs were always present in the urine (personal unpublished data), probably as an organ response to overcome ischemia and reperfusion injury. Of note, no PECs were isolated from residual diuresis prior to transplantation, ruling out the native kidneys as a source of these cells in patients with ESRD. Therefore, we then investigated the presence of uPECs at the time of 6 months protocol biopsy. We hypothesized that in stable kidney allograft recipients the presence of uPECs could be related with classical mechanisms of allograft damage (Yang et al., 2015; Riella et al., 2016; Vanhove et al., 2017). However, no differences were found between uPEC positive and negative patients at cell isolation time point regarding renal function, albuminuria, histological reports and even activation of immune T and B cell responses. This finding modifies previous observations suggesting that it was only possible to isolate uPECs in proteinuric patients (Arcolino et al., 2016) and opens a new focus of study in the field of kidney transplantation. Nevertheless, and differently to patients with glomerulonephritis where the magnitude of the damage is larger resulting in a higher amount of cell loss (Achenbach et al., 2008), the number of cells excreted in urine is very low and uncountable by cytospins, requiring cell culture expansion for analysis.

After we were able to isolate these cells from the urine of stable kidney allograft recipients we sought to determine their functional characteristics. Previous studies demonstrated that these uPECs maintained in culture their clonogenicity and their renal fate *in vitro* as compared to tissue isolated PECs (Lazzeri et al., 2015). Therefore, we confirmed their multilineage differentiation potential, and no previous commitment was observed (*de novo* expression of podocyte and tubular cell markers after differentiation was evidenced). Moreover, as previously reported (Lazzeri et al., 2015) we found that gene expression profile regarding some stem cell recruitment, proliferation, activation, pro-fibrotic and progenitor cell commitment molecules were similar in uPECs and PECs isolated directly from kidney tissue.

Two lines of evidence have been assigned to PECs on glomerular cell balance, supporting a pro-sclerotic vs. a regenerative pattern. On one hand, it was reported that PECs migrate onto the glomerular tuft and participate in glomerular scar formation by extracellular matrix deposition, characteristically proliferating and expressing the activation marker CD44 (Smeets et al., 2014; Roeder et al., 2017), and this was also confirmed in focal segmental glomerulosclerosis in allograft human biopsies (Fatima et al., 2012). On the other hand, different acute podocyte damage models demonstrate disease amelioration concurrent to activation and differentiation of PECs toward podocytes (Lasagni et al., 2015). Taken together, aberrant activated PECs might induce crescents and glomerulosclerosis, but in a situation of podocyte damage might turn into favorable disposition by differentiation into podocytes (Hakroush et al., 2014).

As we did not find any clinical, immunological and histological variable associated with the presence of uPECs at 6 months after kidney transplantation, we tried to characterize PEC features in our cohort of stable patients. Based on previous studies (Fatima et al., 2012; Holderied et al., 2015), we analyzed the sclerosing response from PECs on graft biopsies by staining with the activation marker CD44 and compared extracellular matrix deposition and Banff items for chronic lesions at 6 months protocol biopsies, where we found no differences between groups. Thus, our findings cannot support a direct mechanism of damage from activated PECs in a stable kidney graft. Moreover, as previously pointed out, these results were also supported by gene expression profile, where no significant differences were found between uPECs and PECs obtained from kidney tissue in terms of CD44, αSMA nor collagen IV expression. On the other hand, we observed a significantly increased PEC proliferation in kidney allograft biopsies from patients displaying uPECs. Actually, cell cycle activation determined by Ki67 expression on PECs, and not in the glomerular tuft, from 6 m protocol graft biopsies was higher in the uPEC+ than in the uPEC- group. As these uPECs did not significantly differ to those PECs isolated from kidney tissue regarding the expression of CXCL-12, TLR-2, nor Wnt7b, other pathways might be involved in this cell niche activation. This major finding connecting kidney PECs proliferation and uPECs isolation could be explained because such cells undergo cell cycle activation in order to transdifferentiate and migrate into the glomerular tuft directly from the Bowman’s capsule across the urinary space (Appel et al., 2009; Hackl et al., 2013; Hakroush et al., 2014). This mechanism may justify their excretion and further isolation in urine.

However, this scenario did not explain the foremost PEC activation trigger. Therefore, we tested the hypothesis that an underlying podocyte detachment should be present (Hakroush et al., 2014). Podocyte loss was assessed by urinary podocin (Zhai et al., 2016) and it was significantly associated with the presence of uPECs at 6 months. Interestingly, this state could not be predicted by the pathologic evaluation of the protocol biopsies nor by renal function, proteinuria or immunological effector mechanisms, raising a new approach of podocyte stress (Fulladosa et al., 2003; Kriz and Lemley, 2015). Compensatory glomerular hypertrophy is developed due to reduced nephron number in transplanted kidneys, what directly correlates to allograft outcomes (Fulladosa et al., 2003; Wiggins et al., 2005). Furthermore, it has been recently reported that transplant glomerulopathy is associated with an increased rate of podocyte detachment, a significant reduction in podocyte density and proteinuria (Yang et al., 2015).

We then investigated whether uPECs presence were associated with kidney allograft outcome. Thus, we followed our cohort up to 2 years after transplantation. We found that those uPEC+ patients at 6 months showed a significant eGFR decline and albuminuria at 2 years follow up, in contrast to those uPEC- allografts that maintained a stable eGFR without albuminuria. Then, the isolation of uPECs at 6 months after transplantation appears to be a predictor of worse long-term graft outcomes. uPECs are an early marker of glomerular cell turnover associated to podocyturia, prior to proteinuria and allograft nephropathy. Moreover, we can support this fact on 24 months available protocol biopsies, where we observed a significantly increased mesangial matrix expansion and IgM deposition on those allografts having uPEC+ at 6 months after transplantation. Interestingly, the uPEC+ group showed a trend of PECs proliferation persistence at 2 years as seen by Ki67 staining whereas, again, no activated PECs evaluated by CD44 expression are predominant, suggesting that these cells might be involved in a regenerative intention instead of a pro-sclerotic role, but fail in regenerate chronic podocyte loss as seen in aging models (Berger and Moeller, 2014; Wanner et al., 2014) or increased body size (Berger and Moeller, 2014). In agreement with the 6 months results, no significant differences were found regarding alloimmune response assessed by *de novo* DSA monitoring, previous history of acute rejection as well as inflammation and C4d deposition in 24 months protocol biopsies. These findings suggest again a non-immunological mechanism as the key effector mechanism accounting for podocyturia and PEC proliferation. Recently, Stegall et al. (2018) showed that renal allografts sustain non-immunologic chronic histologic injury by 10 years after transplantation, mainly mesangial sclerosis and glomerulosclerosis, suggesting that new approaches are needed to decrease late injury. Whether this pathogenic mechanism is mainly due to low nephron number (Yang et al., 2015), limited PEC endowment (Berger and Moeller, 2014) or requires combination with additional factors that contribute to glomerular hypertension and podocyte detachment requires further investigation.

Our study has some limitations. Firstly, urine sample contamination may difficult to efficiently assess the presence of uPEC in all cases, but this was overcome with a rigorous collection protocol. Secondly, due to the exploratory nature of the study we did not assess the baseline nephron number. Finally, 24 months protocol biopsy was not available in all patients although clinical and analytical data was obtained to all of them. However, our study has important strengths: the prospective study design, the accurate assessment of the immune response including the protocol biopsies and the innovative approach to ascertain further mechanisms accounting for allograft attrition.

## Conclusion

**FIGURE 6:**
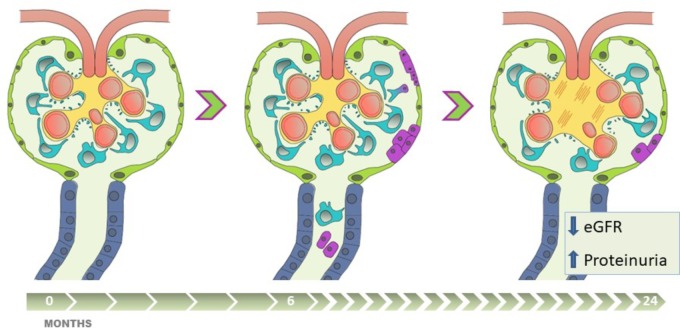
The presence of uPEC in stable kidney transplant recipients anticipates eGFR decline, albuminuria and glomerulosclerosis. Apart from alloimmune damage and glomerular disease recurrence, pathophysiologic mechanisms of chronic allograft damage occur in certain stable-supposed kidney grafts, progressing along critical steps with (1) podocyte stress and detachment, (2) glomerular rearrangement, (3) PECs proliferation and migration into the glomerular tuft, with uPECs as a novel biomarker of this process, (4) subsequent mesangial expansion and extracellular matrix deposition. This damage-response regenerative process, if sustained in time, might fail in preserve allograft function, and is translated into worse long-term results with eGFR decline, a significant albuminuria appearance, and chronic glomerular histological lesions.

The presence of uPECs at 6 months after kidney transplantation translates a hidden damage that cannot be evidenced by renal function and conventional histology. This damage is probably due to podocyte stress that cause podocyte detachment, what becomes a trigger for the proliferation and migration of PECs. However, this mechanism of cell repair is insufficient if the damage is maintained over the time (**Figure 6**). Of note, these cells appear to be a non-invasive biomarker providing contemporary and clinically relevant information. This opens an extensive field of knowledge as a new diagnostic tool and therapeutic target.

## Ethics Statement

This study was carried out in accordance with the recommendations of GCP, Bellvitge Hospital Institutional Review Board. The protocol was approved by the Bellvitge Hospital Institutional Review Board (approval reference PR075/12). All subjects gave written informed consent in accordance with the Declaration of Helsinki.

## Author Contributions

AM, PR, and JC designed the study. AM, EM, and OB were involved in patient follow-up. AM, RG, AS, and EC carried out the experiments. MG performed the pathological evaluation. AM, RG, and JC analyzed the data. AM and RG made the figures. JC, AM, EL, and PR drafted and revised the paper. All authors approved the final version of the manuscript.

## Conflict of Interest Statement

The authors declare that the research was conducted in the absence of any commercial or financial relationships that could be construed as a potential conflict of interest.
